# Cell type and context-specific function of PLAG1 for *IGF2* P3 promoter activity

**DOI:** 10.3892/ijo.2012.1641

**Published:** 2012-09-26

**Authors:** MONIRA AKHTAR, CLAES HOLMGREN, ANITA GÖNDÖR, MATTIAS VESTERLUND, CHANDRASEKHAR KANDURI, CATHARINA LARSSON, TOMAS J. EKSTRÖM

**Affiliations:** 1 Department of Clinical Neuroscience, Karolinska Institutet, Karolinska University Hospital;; 2 Department of Molecular Medicine and Surgery, Karolinska Institutet, CMM L8:01, SE-171 76 Stockholm;; 3 Microbiology and Tumor Center; Karolinska Institutet, SE-171 77 Stockholm;; 4 Department of Genetics and Pathology, Uppsala University, Rudbeck Laboratory, SE-751 85 Uppsala, Sweden

**Keywords:** *H19* insulator, PLAG1, *IGF2*, P3 promoter, chromatin immunoprecipitation, Hep3B, JEG-3

## Abstract

The fetal transcription factor PLAG1 is found to be overexpressed in cancers, and has been suggested to bind the insulin like growth factor 2 (*IGF2*) P3 promoter, and to activate the *IGF2* gene. The expression of *IGF2* has partly been linked to loss of CTCF-dependent chromatin insulator function at the *H19* imprinting control region (ICR). We investigated the role of PLAG1 for *IGF2* regulation in Hep3B and JEG-3 cell lines. Chromatin immunoprecipitation revealed cell type-specific binding of PLAG1 to the *IGF2* P3 promoter, which was substantially insensitive to recombinant PLAG1 overexpression in the endogenous context. We hypothesized that the *H19* chromatin insulator may be involved in the cell type-specific PLAG1 response. By using a GFP reporter gene/insulator assay plasmid construct with and without the *H19* ICR and/or an SV40 enhancer, we confirm that the effect of the insulator is specifically associated with the activity of the *IGF2* P3 promoter in the GFP reporter system, and furthermore, that the reporter insulator is functional in JEG-3 but not in Hep3B cells. FACS analysis was used to assess the function of PLAG1 in low endogenously expressing, but Zn-inducible stable PLAG1 expressing JEG-3 cell clones. Considerable increase in *IGF2* expression upon PLAG1 induction with a partial insulator overriding activity was found using the reporter constructs. This is in contrast to the effect of the endogenous *IGF2* gene which was insensitive to *PLAG1* expression in JEG-3, while modestly induced the already highly expressed *IGF2* gene in Hep3B cells. We suggest that the PLAG1 binding to the *IGF2* P3 promoter and *IGF2* expression is cell type-specific, and that the PLAG1 transcription factor acts as a transcriptional facilitator that partially overrides the insulation by the *H19* ICR.

## Introduction

The insulin like growth factor (IGF) system is composed of two related growth factors (IGF-I and IGF-II) and a group of IGF binding proteins (IGFBPs). This system has important roles in human development and maintenance of normal functions of many cells of the mammalian organism. In human, both IGF-I and IGF-II are produced in multiple tissues throughout life and have potentially divergent roles in pathophysiology. IGF-II has various roles in pathological conditions and a particularly prominent role in tumor development. This association between cancer and IGFs has long been a subject for investigation (1). Dysregulation of *IGF2*, its receptors (the type 1 and 2 IGFR), and IGFBPs provide part of the underlying mechanism for uncontrolled increase in cellular proliferation, as is evident in cancer. The *IGF2* locus shows loss of imprinting (LOI) in several types of cancer (reviewed in ref. 2) and it was shown to be a marker for colorectal carcinoma risk (3).

Genome wide epigenetic marks are maintained with high fidelity in normal cells but frequently become destabilized in human cancer (4,5). This is exemplified by the observations that the normally silenced state of the maternally inherited *IGF2* allele is lost in several cancer forms, such as Wilms’ tumor (6) and colon cancer (7), resulting in LOI and an activation of biallelic expression patterns. The finding that *IGF2* LOI in peripheral blood cells is the earliest predictive marker for colon cancer reinforces the notion that constitutive epigenetic lesions predispose for cancer (3). The normally repressed states of the paternal *H19* and the maternal *IGF2* alleles are coordinated by the differentially methylated imprinting control region (ICR or DMR) in the 5′-flank of the *H19* gene (8). This feature involves a methylation-sensitive, long-range chromatin insulator (9), that via target sites for the 11 zinc finger protein CTCF represses the maternal *IGF2* allele in mammals (10,11).

It has further been shown that in mouse, this insulator region is in physical contact with the promoter region of the *Igf2* gene (10,12) and that cohesin is maintaining the insulation properties in the *H19/IGF2* locus (13). In essence, the binding of CTCF to the ICR, prevents the *Igf2/IGF2* gene from being in close proximity to the enhancers located in the 3′-flank of the *H19* gene, while they can interact with the *H19* promoter. The absence of CTCF binding lets the same enhancers interact with the *Igf2/IGF2* gene. The fact that the binding of CTCF to the ICR is methylation sensitive further strengthens the notion that the methylation status of the ICR is the most crucial key for proper maintenance of the mono-allelic expression patterns of these genes.

Reports have shown that the CTCF target sites within the *H19* ICR are *de novo* methylated in a wide range of human cancers (14,15), which would lead to prevention of CTCF binding and loss of insulator function at the maternal allele, followed by *IGF2* allelic reactivation. This phenomenon can, however, not explain the appearance of LOI of *IGF2* in correlation with hypomethylation at the maternal ICR which has been reported in colon cancer (16) and bladder cancer (17). These findings suggest alternative, ICR independent, mechanisms that can reactivate the silenced *IGF2* gene on the maternal allele.

*PLAG1* is a proto-oncogene discovered in pleomorphic adenoma of the salivary glands (18). It has been described as a seven zinc finger transcription factor which is developmentally regulated and expressed during fetal development in several tissues, but is downregulated after birth. One reported mechanism for deregulation of *PLAG1* is promoter swapping with the β-catenin gene, resulting in ectopic expression of *PLAG1* in pleomorphic adenoma of the salivary glands (18). PLAG1 has been shown to have oncogenic capacity and to potently trigger *IGF2* expression by its binding to the P3 promoter as shown by electromobility shift assay (EMSA) (19). In addition *PLAG1* has been shown to be overexpressed in several other tumor types, such as hepatoblastoma (20), lipoblastoma (21) and acute myeloid leukemia (22).

Here, we set out to investigate the role of PLAG1 in the regulation of *IGF2* in an insulator-reporter system as well as at the endogenous level in cell lines. The findings provide evidence that PLAG1 can activate *IGF2* P3 promoter-dependent expression, despite the presence of the ICR in a reporter system. The endogenous sensitivity of *IGF2* to PLAG1 displayed a cell type-specific response, and the *IGF2* P3 promoter revealed clear differential capacity to bind the PLAG1 transcription factor which was however, not reflected by the *IGF2* transcription.

## Materials and methods

### Cell lines and stably transfected cell clones

JEG-3 (human choriocarcinoma) and Hep3B (human hepatocellular carcinoma) cell lines were maintained as previously described (23). For stable transfection, 2 *μ*g of the pSAR-PLAG1 (19) construct and 0.1 *μ*g of the pBK-CMV plasmid (neo +) was co-transfected into JEG-3 cells using Fugene 6 (Roche) according to the protocol recommended by the manufacturer, followed by neomycin selection (500 *μ*M) for 4 weeks. Resistant cell clones were picked, expanded and analysed for zinc-inducible (100 *μ*M ZnCl_2_) *PLAG1* expression using quantitative real-time PCR (qRT-PCR). One zinc responsive cell clone which had undetectable PLAG1 mRNA in the absence of zinc was selected for further experimental procedures.

### Quantitative real-time PCR analysis of IGF2 and PLAG1

qRT-PCR was performed to evaluate the *IGF2* and *PLAG1* mRNA levels in wild-type Hep3B and JEG-3 cells. Total-RNA was extracted using Trizol and phenol-chloroform, quantified by Nanodrop, and 6–9 *μ*g of RNA was treated with DNase I (6–7 units, at 37°C for 30 min). DNase I treated RNA (1 *μ*g) was used for cDNA synthesis with iScript cDNA synthesis kit (Bio-Rad) in 20 *μ*l reactions. Reverse transcription was performed in 20 *μ*l reactions following the protocol from the manufacturer. A total of 2 *μ*l of product was used for PCR amplification of individual transcripts from *IGF2* and *PLAG1* in an Applied Biosystem 7500 Fast Real-Time PCR instrument using the iScrip SYBR-Green kit (Bio-Rad). The primers for *IGF2* are F: 5′-AAC TGG CCA TCC GAA AAT AGC and R: 5′-TTT GCA TGG ATT TTG GTT TTC AT. For *PLAG1* primers sequences are F: 5′-CAC ACA GGA GAG AGG CCC TAC and R: 5′-CAC AAT AAT TAC ACT TGT. Thermo cycling conditions were 50°C for 2 min and 95°C for 5 min, followed by 40 cycles of 95°C for 20 sec and 57° or 67°C for 30 sec for *IGF2* and *PLAG1*, respectively. The GAPDH mRNA was used for normalization to correct for the amount of RNA loaded in each sample. Primer sequences are F: 5′-GAA GGT GAA GGT CGG AGT C and R: 5′-GAA GAT GGT GAT GGG ATT TC. Thermo cycling conditions were 50°C for 2 min and 95°C for 5 min, followed by 40 cycles of 95°C for 20 sec and 60°C for 30 sec.

### Semi-quantitative RT-PCR

Semi-quantitative RT-PCR of PLAG1 transcripts was performed in JEG-3 stable clones before and after Zn-induction using the primers mentioned above. Thermo cycling conditions were 94°C for 5 min, followed by 30 cycles of 94°C for 30 sec, 59°C for 50 sec and 70°C for 50 sec and final extension at 72°C for 10 min.

### Transient transfection and expression of IGF2 P3 driven GFP reporter constructs

The *IGF2* P3 promoter was cloned as a *Bam*HI-*Sal*I fragment into the *Bgl*II-*Sal*I site of the pd2EGFP-1 vector (Clontech) to create the pd2EGFP1-IGF2-P3-GFP intermediate vector. The IGF2-P3-GFP cassette was excised from the intermediate vector by digesting with *Eco47*III-*Ssp*I and cloned into a *Bstz17*I digested pREP4 H19A vector (23) to create the pA-GFP plasmid. The pB-GFP was obtained by cloning a 3.3 kb *Kpn*I-*Xho*I mouse *H19* ICR fragment into pA-GFP. The pO-GFP plasmid was generated by deleting the SV40 enhancer from the pA-GFP plasmid by *Cla*I digestion followed by re-ligation. pRep9RFP was created by cloning a *Kpn*I-*Xho*I fragment containing the RFP-gene (derived from pDsRed1, Clontech) into the pRep9 vector (Invitrogen).

Equimolar amounts (3 *μ*g) of the GFP constructs were co-transfected with 0.25 *μ*g pDsRed2-C1 (Clonetech) or 1 *μ*g pRep9RFP into JEG-3 and Hep3B cells using Fugene 6 (Roche) according to the protocol recommended by the manufacturer. GFP expression was analysed using confocal microscopy (Leica DM Irbe) and flow cytometry (BD FACSCalibur), whereby the expression levels of GFP were estimated as a ratio between GFP and RFP expression (constant).

### Chromatin conformation analysis

Nuclei from Hep3B and JEG-3 cells, transfected with the pB-GFP construct, were isolated and DNase I treated as previously described (23). DNase I treated DNA (20 *μ*g) was digested with *Stu*I and analysed in 1.7% agarose gels followed by Southern blotting according to standard procedures. The chromatin conformations were analysed by indirect end-labelling as has been accounted for before (23). The chromatin conformation was analysed by using a 170 bp *Stu*I-*Eco*RI fragment spanning the upstream region outside of the ICR as previously described (9).

### Transient transfection of PLAG1

Hep3B and JEG-3 cells were transiently transfected with 3 *μ*g of the PLAG1 expression vector pCAGGS-PLAG1 (20) on 60-mm plates for 72 h. As negative control, mock transfection with a β-galactosidase containing plasmid was done in parallel. Transfection was performed using the Lipofectamine and Plus reagent transfection kit (Invitrogen) according to the manufacturer’s protocol.

### Chromatin immunoprecipitation (ChIP) analysis

Binding of PLAG1 to the *IGF2* P3 promoter was investigated in Hep3B and JEG-3 cells using Chromatin Immunoprecipitation (ChIP). ChIP was performed from total cellular extracts according to the Quick and Quantitative Chromatin Immunoprecipitation (Q2 ChIP) method (described in ref. 24). After trypsinization cells were cross-linked with 1% formaldehyde and chromatin was sheared by Bioruptor 10 times (10 sec each) to fragments of 200 to 1,000 base pairs and cleared for cellular debris by sedimentation. The supernatant was used for immunoprecipitation of specific protein-DNA complex using a PLAG1 antibody (Sigma, cat no. AV 38177) and normal rabbit IgG coupled to Dyna beads protein-G (Invitrogen) at 4°C overnight. IgG was used as a control for non-specific binding due to Fc receptor binding or other protein-protein interactions. ChIP complexes were washed with RIPA buffer and subsequently cross-links were reversed, proteins digested and DNA eluted in elution buffer. DNA was purified with PCR Purification Kit (Qiagen). The immunoprecipitated DNA was then quantified by SYBR-Green qPCR using primers flanking the *PLAG1* consensus binding site in the *IGF2* P3 promoter region (20). Primer sequences are F: 5′-CTGCCTGCCCGGAGAC and R: 5′-CATGCTGAATGCCCGTTCT. Thermo cycling conditions were 50°C for 2 min and 95°C for 5 min, followed by 40 cycles of 95°C for 20 sec and 65°C for 30 sec.

## Results

### Expression of IGF2 and PLAG1 in cell lines

**Figure 1:**
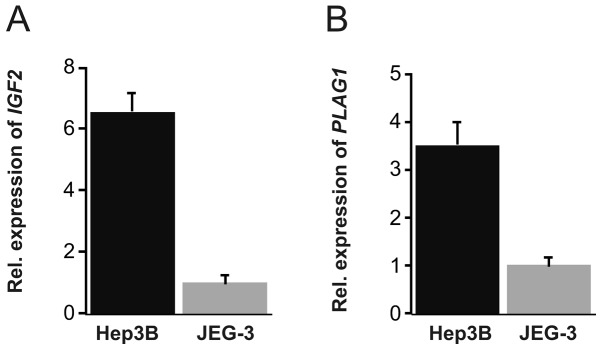
Detection of *IGF2* and *PLAG1* transcripts in Hep3B and JEG-3 cells. (A) qRT-PCR analysis of the relative expression level of *IGF2* mRNA. (B) Real-time qPCR analysis of *PLAG1* mRNA. All experiments were run in triplicate. Error bars denote standard error of the mean.

**Figure 2:**
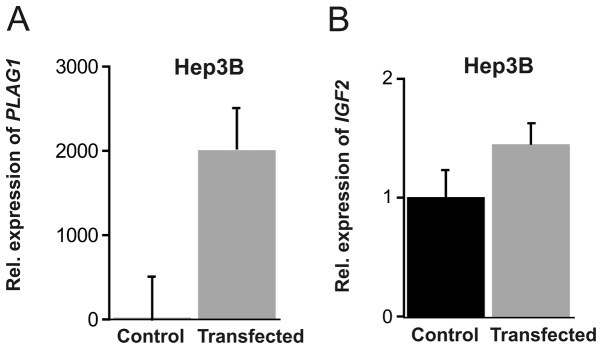
Detection of *IGF2* and *PLAG1* expression in Hep3B cells following transfection with the *PLAG1* expression vector, pCAGGS-PLAG1. Cells were harvested 72 h post-transfection. (A) *PLAG1* transcripts relative to mock transfected control. (B) *IGF2* transcript after *PLAG1* overexpression, relative to mock transfected control. The experiments were run in triplicate. Error bars denote standard error of the mean.

**Figure 3:**
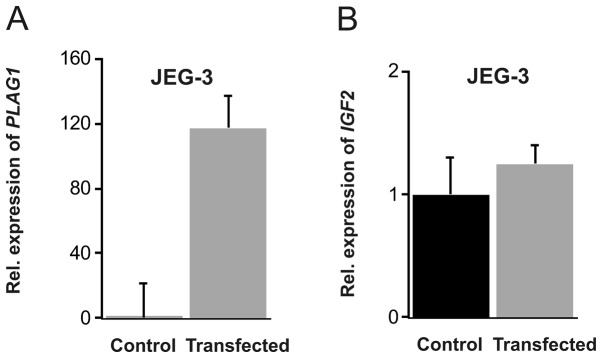
Detection of *IGF2* and *PLAG1* expression in JEG-3 cells following transfection with the *PLAG1* expression vector, pCAGGS-PLAG1. Cells were harvested 72 h post-transfection. (A) *PLAG1* transcripts relative to mock transfected control. (B) *IGF2* transcripts after *PLAG1* overexpression, relative to mock transfected control. The experiments were run in triplicate. Error bars denote standard error of the mean.

Since Hep3B and JEG-3 cells were used as model systems, we assessed the endogenous mRNA levels of *PLAG1* and *IGF2* by qRT-PCR using the housekeeping gene GAPDH as normalization factor. Substantially higher level of *IGF2* (around 6-fold) was found in Hep3B as compared to JEG-3 cells (Fig. 1A). *PLAG1* expression level was approximately 3 times higher in Hep3B compared to JEG-3 cells (Fig. 1B). We therefore investigated whether *PLAG1* expression may be an important player for higher expression of *IGF2* in Hep3B cells, and if transient over-expression of *PLAG1* could elevate *IGF2* transcription. For this purpose we transfected Hep3B and JEG-3 cells with a *PLAG1* expression plasmid. In Hep3B cells, a monumental 2,000-fold induction of *PLAG1* mRNA was achieved (P=0.0026), together with a modest increase of *IGF2* expression (Fig. 2). In JEG-3 cells, the transfection was less efficient in comparison to Hep3B, but a robust 130-fold increase in *PLAG1* mRNA was achieved (P=0.017). This did not, however, result in any enhanced *IGF2* expression (Fig. 3).

### ChIP analysis

**Figure 4:**
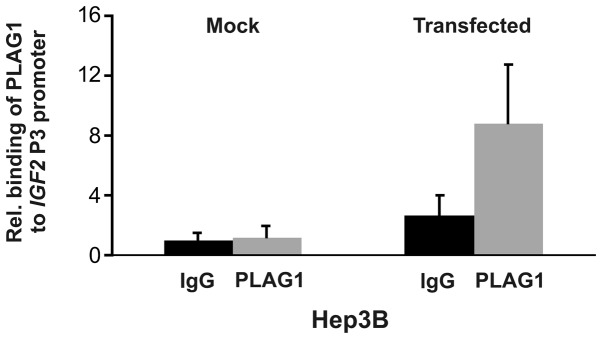
Analyses of PLAG1 binding at the *IGF2* P3 promoter in Hep3B cells as determined by ChIP. Chromatin fragments were immunoprecipitated with anti-PLAG1 antibody and IgG as a control, and quantified by SYBR-Green qPCR. The ChIP analyses were run in triplicate and error bars denote standard error of the mean.

The finding that ectopic PLAG1 expression slightly induced endogenous *IGF2* expression in already high *IGF2* expressing Hep3B cells, but not in JEG-3 cells, prompted us to analyze binding of PLAG1 to the *IGF2* P3 promoter using chromatin immunoprecipitation. Mock transfected Hep3B cells did not display increased binding of PLAG1 to the P3 promoter. However, transient transfection with PLAG1 resulted in increased binding (Fig. 4). The situation for JEG-3 cells was dramatically different. No binding of PLAG1 to the P3 promoter could be detected in non-transfected, or PLAG1 transfected cells (data not shown), consistent with the low endogenous *IGF2* expression and the inability to boost this by additional expression of PLAG1 (Fig. 3).

### IGF2 P3 promoter reporter expression in the presence of a functional insulator

Our results on the relatively weak and cell type-specific endogenous relationship between *IGF2* expression and PLAG1 binding to the P3 promoter, prompted us to turn to a simpler system that allowed us to separate the P3 promoter from the important H19 3′-enhancers. We hypothesized that Hep3B and JEG-3 cells have different sensitivity to the *H19* upstream insulator in the regulation of *IGF2* P3 promoter and that *PLAG1* might be involved in this.

**Figure 5:**
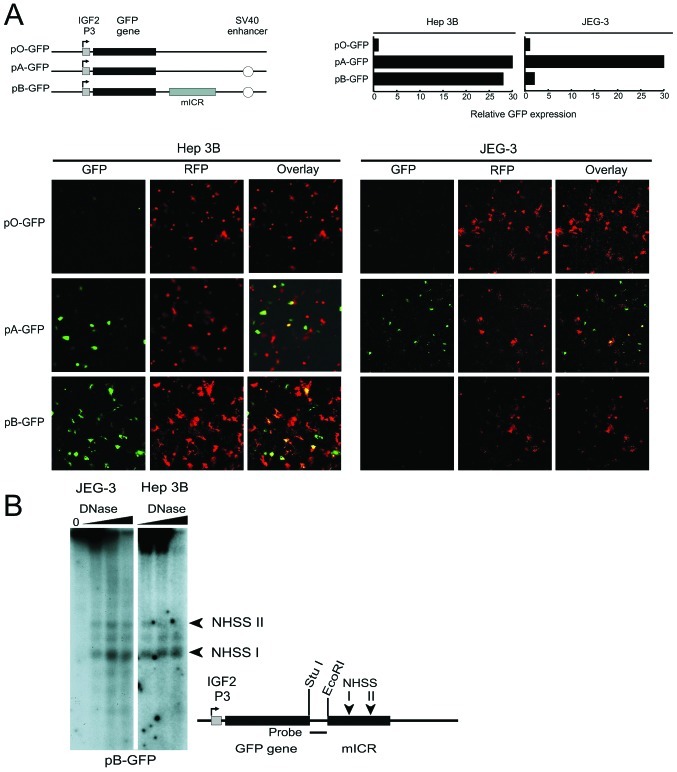
The insulator function of the ICR can be bypassed in human cancer cells and correlates to levels of PLAG1 expression. (A) Schematic illustrations of the GFP reporter constructs used in the insulator assays. The analysis of GFP expression was performed using flow cytometry (upper right) where the bars depicts relative GFP expression after correlation to RFP expression (pRep9RFP) and with confocal microscopy where the panels show the GFP expression pattern of the different reporter genes in a subset of cells in Hep3B and JEG-3. The left sections show GFP expression in the construct. The middle section of each panel shows the expression of the transfection control construct, DsRed2. The right sections show an overlay, where the distinctive GFP and RFP expression pattern can be determined. (B) The chromatin conformation of the transfected ICR is similar in both GFP expressing and non-expressing cells. DNase I-treated nuclei from JEG-3 and Hep3B transfected with pB-GFP, was digested with *Stu*I. The nuclease hypersensitive sites (NHSS I and II) correspond to CTCF target sites [Kanduri *et al*(23)] and are identical in both cell lines.

In order to investigate the insulator effect of the ICR, we developed a GFP based assay system (Fig. 5A) where we could analyze the effects of insulator function in real-time using confocal microscopy as well as expression analysis using FACS. JEG-3 and Hep3B cells were simultaneously transfected with the P3/GFP episomal constructs (Fig. 5A) and analyzed at 48h post-transfection. In JEG-3 cells the GFP expression was low in the enhancer-free promoter construct, pO-GFP, and substantially higher in the SV40 enhancer containing construct, pA-GFP (Fig. 5A). The construct which also contains an insulator, pB-GFP, showed an almost total enhancer insulation of GFP expression, as expected (Fig. 5A). The Hep 3B cell line, however, displayed a different expression pattern. As in JEG-3 cells, GFP expression was low in the enhancer-less promoter construct pO-GFP and robust in the enhancer-containing pA-GFP construct. However, in contrast to JEG-3 cells, this high expression was not attenuated in Hep3B for the insulator containing construct pB-GFP (Fig. 5A). The possible explanation that the insulator was non-functional by the absence of binding by CTCF at the ICR, was ruled out, since the chromatin conformations at the CTCF target-sites of the transfected *H19* ICR were virtually identical, in JEG-3 cells and Hep3B cells (Fig. 5B).

To analyse if this aberrant expression of GFP was promoter-dependent, the *IGF2* P3 promoter was replaced with the *H19* promoter in the otherwise identical constructs. FACS analyses verified that the insulator function was restored in Hep3B with these constructs, suggesting that the loss of insulator function is *IGF2* P3 promoter-dependent (data not shown). This finding is further supported by previous observations where the ICR has been shown to be a potent insulator of the *H19* promoter in Hep3B (23). Since it was previously shown that PLAG1 can upregulate *IGF2* expression and that binding to the P3 promoter could occur in an EMSA (19), together with our expression and ChIP results above, we hypothesized that PLAG1 might be involved in the cell type-specific expression of *IGF2* that involves the *H19* ICR.

### PLAG1 expression attenuates the insulator function of the H19 ICR

**Figure 6:**
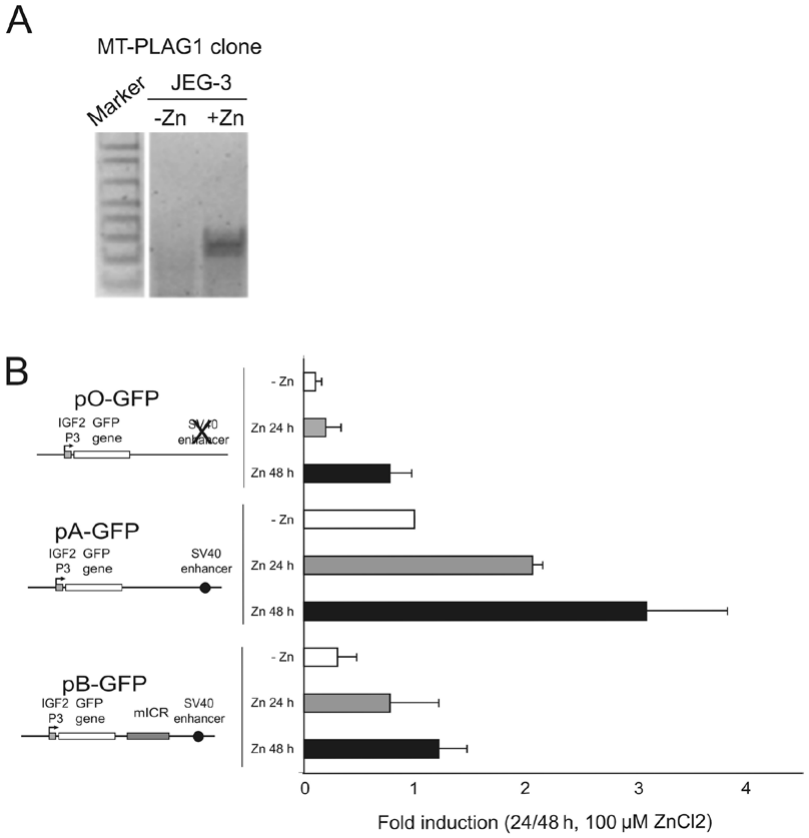
Expression analyses of JEG-3 cells in a stable MT-PLAG1 clone and subsequent transient IGF2-P3-GFP transfection. (A) Semi-quantitative RT-PCR analysis showing increased *PLAG1* expression in the Zn-treated MT-PLAG1 JEG-3 clone (+Zn) as compared to untreated cells (−Zn). B. The MT-PLAG1 JEG-3 cell-clone was transfected with *IGF2*-P3-GFP reporter constructs and analysis of GFP expression was performed using flow cytometry. The bars depict relative GFP expression after correlation to RFP expression (DsRed2) and indicate fold induction of GFP expression with non-induced (−Zn) and induced (+Zn) *PLAG1* expression with different reporter constructs. The expression levels of GFP was analysed after both 24 and 48 h, showing an increased level of GFP expression after 48 h of induction. The expression level of the non-induced pA-GFP construct is set at 1 and is referred to as the basic state of enhanced expression. The error bars denote the SEM of three independent experiments.

Since the expression of *PLAG1* was significantly lower in JEG-3 than in Hep3B (Fig. 1B), and the GFP-reporter construct displayed insulator activity in JEG-3 but not Hep3B cells, we examined whether increased *PLAG1* expression in JEG-3 would result in a Hep3B-like situation with the GFP reporter construct. We created a zinc-inducible *PLAG1* cell clone derived from JEG-3 by the stable incorporation of the pSAR-*PLAG1* construct (19). Several cell clones were analysed and one zinc-inducible clone, but with undetectable *PLAG1* expression in the absence of ZnCl_2_, was selected for the subsequent analyses. Upon induction with ZnCl_2_ the level of PLAG1 expression increased substantially in this clone (Fig. 6A). The episomal GFP constructs (Fig. 5A) were co-transfected with RFP control constructs into the *PLAG1* inducible JEG-3 cell line, followed by qRT-PCR analysis of GFP expression. Upon Zn-induction of *PLAG1*, the expression of the GFP reporter gene was considerably elevated in all constructs (Fig. 6B). By employing the enhancer- and insulator-free construct (pO-GFP) it was shown that after 48 h of induction, PLAG1 can elevate the levels of transcription from the P3 promoter to levels similar to the enhancer driven construct (pA-GFP) in low-*PLAG1* expressing cells. In the presence of the enhancer, PLAG1-induction dramatically increased the P3 driven GFP expression. Interestingly, PLAG1 could also induce high expression in the insulator-containing pB-GFP construct. Although the insulator still appears to maintain an ability to attenuate enhancer-promoter interaction in the presence of *PLAG1* overexpression (Fig. 6B, pB-GFP construct, Zn 48 h), the level of this expression is higher than both the induced pO-GFP construct (Fig. 6B, +Zn 48 h), and more importantly, even from the non-induced enhancer-containing construct lacking an insulator, pA-GFP (Fig. 6B, −Zn). Thus, after 48 h of PLAG1 induction, the levels of P3-driven *GFP* in the presence of insulator slightly supersede non-induced cells containing the insulator-free construct. This finding suggests that the stringency of the system normally regulated by insulator-action is partially lost in JEG-3 cells when *PLAG1* is overexpressed.

## Discussion

In the current study we report on a novel aspect of cell line specific *IGF2* regulation involving the PLAG1 transcription factor, its binding to the P3 promoter region, and the possible consequence of *PLAG1* expression in the context of promoter/enhancer interaction. Although, it has been shown previously by EMSA that the *IGF2* P3 promoter contains PLAG1 consensus-binding sites (19), the binding of PLAG1 to the *IGF2* P3 promoter in live cells has not been reported before. We therefore investigated this by ChIP analysis. The JEG-3 and Hep3B cells were analyzed in order to verify the notion that PLAG1 is important for activity of the *IGF2* P3 promoter. We show here that *PLAG1* overexpression only increase binding of PLAG1 to the P3 promoter in Hep3B cells, leading to moderate increase of the endogenous *IGF2* expression in Hep3B, while neither promoter binding nor expression were affected in JEG-3 cells. On the other hand, PLAG1 stimulated P3 promoter driven GFP transcription in JEG-3 cells as well as overcame, at least in part, the *H19* insulator in a GFP-plasmid reporter system.

Transcriptional insulators are specialized cis-acting elements that isolate promoters from positive and negative influences by distal enhancers. The *H19* upstream insulator is also an ICR, which regulates the allele-specific transcription of the *IGF2* gene in a parent of origin-dependent manner. This is achieved by methylation of the ICR on the paternal allele leading to hindrance of the insulator protein CTCF and loss of communication between the shared *H19/IGF2* enhancer downstream of *H19* and the *IGF2* promoter(s) (12). Methylation of the ICR also spread into the *H19* promoter leading to transcriptional silencing. Conversely, the unmethylated ICR on the maternal allele binds CTCF and then functions as an enhancer-blocking insulator that prevents expression of the maternal *IGF2* allele.

Our findings suggest a new role for PLAG1 in *IGF2* overexpression in cancer. Not only does PLAG1 expression affect the activity of the promoter, it also appears to increase the effect of enhancer activated transcription. The function of PLAG1 as a regular transcription factor and proto-oncogene has been reported previously, and there is previous evidence to show that it binds to a consensus binding site in the *IGF2* P3 promoter and activates its transcription *in vitro*. We propose, however, that PLAG1 has additional, more complex functions, one of which may be to act as a promoter/enhancer facilitator. The reporter constructs used in this study were designed to interrogate the influence of insulator function of the *H19* ICR on *IGF2* P3 promoter activity in cells with different levels of *PLAG1* expression. We observed that JEG-3 cells are relying on an exogenous *PLAG1* expression to overcome the silencing effect of the insulator in the construct, although they do transcribe low levels of *PLAG1* mRNA. Hep3B cells on the other hand, which have a considerably higher endogenous *PLAG1* expression, are already insensitive to the insulator. In this connection, it is important to note that the chromatin structure of the ICR in the episomal construct, was found to be similar in both JEG-3 and Hep3B cells (Fig. 5B), indicating presence of binding by CTCF, and thus promoting insulation, in both cases. In this scenario the induction of PLAG1 was apparently able to partially overcome an active insulation since the ICR containing construct (pB-GFP) showed a substantially higher *GFP* expression than the enhancer-free and ICR-free construct (pO-GFP), after PLAG1 induction (Fig. 6B). The inability to completely overcome insulation was evident, however, since *PLAG1* expression in the ICR containing construct did not reach the *GFP* expression level of the ICR-less/enhancer containing construct (pA-GFP).

It is unknown why the low endogenous *PLAG1* expression in JEG-3 cells does not affect the expression of the GFP reporter. It is possible that a threshold concentration of PLAG1 is required to push a transcription factor complex towards assembly.

Considering that in the GFP reporter construct, PLAG1 is able to influence the *IGF2* P3 promoter activity in JEG-3 cells, it is challenging to understand why the functional response to PLAG1 is different from that in the endogenous P3 promoter. Maybe PLAG1 binding in the endogenous *IGF2* P3 promoter requires additional factors, absent in JEG-3 cells but present in Hep3B. Although we did demonstrate that highly overexpressed PLAG1 actually increased binding to endogenous P3 in Hep3B cells, only moderate activation of *IGF2* transcription was achieved and non-transfected cells did not demonstrate substantial endogenous PLAG1 binding to the P3 promoter suggesting that other factors, not excluding chromatin structure, are important for the endogenous *IGF2* transcription. This was in contrast to the JEG-3 cells where neither binding nor activation could be demonstrated despite substantial PLAG1 expression. These cells are apparently not geared at all for PLAG1 directed *IGF2* expression, and since the GFP-reporter construct was PLAG1 inducible, chromatin structure in the endogenous gene may be an important factor. The lack of additional *IGF2* activation in Hep3B cells after *PLAG1* overexpression may also be due to an already saturated promoter by other permissive transcription factors, or lack of required additional factors. The PLAG1 protein may also be sumoylated and thus inactivated, as was shown by others (25). To our knowledge, whether or not sumoylation of PLAG1 influences its binding to the *IGF2* P3 promoter has not been demonstrated. It is also possible that overexpression of *PLAG1* does not stimulate the promoter P3-dependent transcription of *IGF2* in JEG-3 cells, due to methylation mediated inactivation of the P3 promoter or the *H19* DMR. These questions are currently being analyzed.

Although it would be a possibility that PLAG1 interferes with allele-specific expression of *IGF2* by interfering with the ICR, we did not study this issue here. In addition, Declercq *et al* concluded that only the paternal *Igf2* allele is involved in the PLAG1 induced formation of pleomorphic adenomas of the salivary glands, in a transgenic mouse model (26). This suggests that although PLAG1 in certain contexts may interefere with the H19 ICR, it is not involved in the imprinting mechanism.

In conclusion, we show here that the role of PLAG1 in activation of *IGF2* is context-specific in that it readily activates an episomal reporter driven by the *IGF2* P3 promoter, it is cell type-specific in its sensitivity to promoter enhancer insulation and in the endogenous context, and that both binding to the *IGF2* P3 promoter and endogenous transcription activation is cell type-specific. We also suggest that PLAG1 may act as a facilitator since it partially overcomes the insulator function of the *H19* DMR, at least *in vitro*.
